# A primary undifferentiated pleomorphic sarcoma of the lumbosacral region harboring a LMNA-NTRK1 gene fusion with durable clinical response to crizotinib: a case report

**DOI:** 10.1186/s12885-018-4749-z

**Published:** 2018-08-22

**Authors:** Ning Zhou, Reinhold Schäfer, Tao Li, Meiyu Fang, Luying Liu

**Affiliations:** 1 Department of Abdominal Radiotherapy, Zhejiang Cancer Hospital, Hangzhou, Zhejiang 310022 People’s Republic of China; 2 Comprehensive Cancer Center, Charité Universitätsmedizin Berlin, Charitéplatz 1, D-10117 Berlin, Germany; 3 Department of Bone and Soft-tissue Surgery, Zhejiang Cancer Hospital, Hangzhou, Zhejiang 310022 People’s Republic of China; 4 Department of Integration of Traditional Chinese and Western Medicine, Zhejiang Cancer Hospital, Hangzhou, Zhejiang 310022 People’s Republic of China

**Keywords:** Undifferentiated pleomorphic sarcoma, Spindle cells, Lumbosacral, LMNA-NTRK1 gene fusion, Crizotinib therapy

## Abstract

**Background:**

High-grade spindle cell sarcomas are a subtype of rare, undifferentiated pleomorphic sarcomas (UPSs) for which diagnosis is difficult and no specific treatment strategies have been established. The limited published data on UPSs suggest an aggressive clinical course, high rates of local recurrence and distant metastasis, and poor prognosis.

**Case presentation:**

Here we present the unusual case of a 45-year-old male patient with a lumbosacral UPS extending into the sacrum. An initial diagnosis of a low-grade malignant spindle cell tumor was based on a tumor core biopsy. After complete extensive resection, the diagnosis of an UPS of the lumbosacral region was confirmed by excluding other types of cancers. Despite treatment with neoadjuvant radiotherapy, extensive resection, and adjuvant chemotherapy, the patient presented with multiple pulmonary metastases 3 months after surgery. The patient then began treatment with crizotinib at an oral dose of 450 mg per day, based on the detection of a LMNA-NTRK1 fusion gene in the tumor by next-generation sequencing. Over 18 months of follow-up through July 2018, the patient maintained a near-complete clinical response to crizotinib.

**Conclusions:**

The LMNA-NTRK1 fusion was likely the molecular driver of tumorigenesis and metastasis in this patient, and the observed effectiveness of crizotinib treatment provides clinical validation of this molecular target. Molecular and cytogenetic evaluations are critical to accurate prognosis and treatment planning in cases of UPS, especially when treatment options are limited or otherwise exhausted. Molecularly targeted therapy of these rare but aggressive lesions represents a novel treatment option that may lead to fewer toxic side effects and better clinical outcomes.

**Electronic supplementary material:**

The online version of this article (10.1186/s12885-018-4749-z) contains supplementary material, which is available to authorized users.

## Background

Undifferentiated pleomorphic sarcoma (UPS), which is also referred to as malignant fibrous histiocytoma (MFH) according to the 2002 World Health Organization classification, is a rare and aggressive type of mesenchymal malignancy with no definitive cell of origin or specific recurrent genetic hallmarks. Extensive immunohistochemical characterization is required to differentiate UPS from other tumors. While UPS can occur throughout the body, these tumors are commonly found in the extremities and in the retroperitoneum [1, 2], and superficial lesions (subcutaneous) are rare. High-grade spindle cell sarcomas are one subtype of UPSs that is particularly challenging to accurately diagnose and effectively treat. The current 5-year overall survival rate for patients with UPSs is only 65–70%, highlighting the need for more effective treatment options [3].

At present, UPSs should be treated according to current guidelines for soft tissue sarcoma (STS), because no standard treatment strategy specific for UPSs has been established. Extensive excision and radiotherapy remain the cornerstones of treatment for non-metastatic tumors. With the majority of these tumors being high grade at diagnosis, localized treatments commonly result in poor local control and poor survival. Perioperative chemotherapy was recently reported to be beneficial in terms of overall survival [4], and doxorubicin as a single agent or in combination with ifosfamide is the first choice of chemotherapy in cases of UPS metastasis. A more complete understanding of the molecular characteristics and cytogenetics of these tumors will aid in the differentiation of sarcoma subtypes and development of specifically targeted therapies. Here we report a rare case of UPS in the lumbrosacral region and review the diagnostic procedures applied in this case as well as the treatment decisions and outcomes.

## Case presentation

A 45-year-old male patient presented with a complaint of progressive pain and soreness in the lumbosacral region persisting for more than 3 months. The pain radiated to the left thigh and perineum but did not affect walking. Magnetic resonance imaging (MRI) and computed tomography (CT) scans with and without intravenous contrast showed a tumor mass adjacent to the left side of the fifth lumbar spinous process. The tumor was located in the lower left part of the erector spinae and extended onto the fifth lumbar vertebra, the first sacral vertebra, and the iliac wing. Positron emission tomography with CT (PET/CT) showed a hypermetabolic lesion in the erector spinae adjacent to the left side of the fifth lumbar spinous process. No sites of regional or distant metastases were found. A core biopsy of the tumor mass revealed spindle-shaped cells with infiltrating inflammatory cells. Together the morphological and immunohistochemical features indicated a low-grade inflammatory myofibroblastic tumor. The expression profile based on immunostaining was as follows: overall positive for vimentin, CD34, ALK (SP8), and p53; focally positive for smooth muscle actin (SMA); sporadically positive for S-100; partially positive for CD68; and negative for cytokeratin (CK) (AE1/AE3), desmin, and CD117. The Ki-67 nuclear labeling index was 10%.

The patient reported no other symptoms. Physical examinations revealed no neuro-pathological signs or symptoms. He denied smoking, alcohol, or illicit drug usage. He also denied recent radiation or toxin exposure. He had no history of unintentional weight loss, fever, or chills. He had no family history of malignant or other chronic diseases, with the exception of a sister who had breast cancer.

**Fig. 1:**
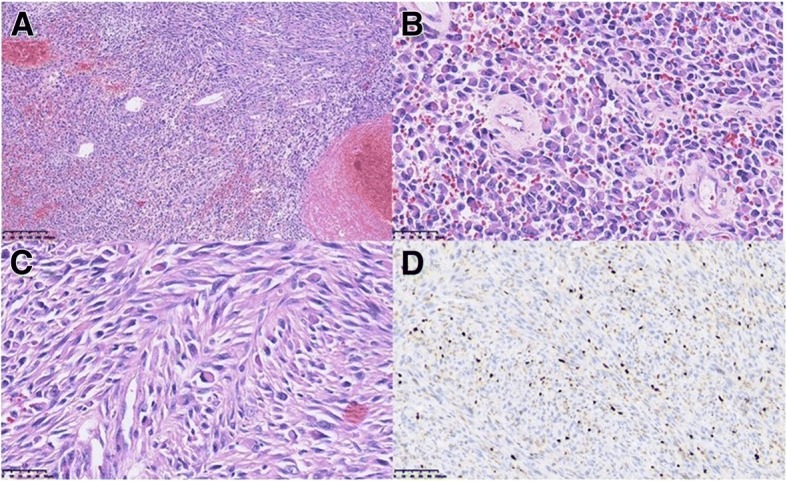
Histopathological staining of surgically resected tumor tissue. Pathology revealed high-grade spindle cell sarcoma consistent with UPS. **a** Hematoxylin and eosin (H&E); magnification, 100×. **b** H&E, 400×. **c** H&E, 400×. **d** Ki-67, 200×. Brown nuclear staining for this proliferation marker is seen in many tumor cells

The treatment plan of the case was discussed by our multi-disciplinary team including experts from orthopedics, neurosurgery, chemotherapy, radiotherapy, pathology, and radiology. Considering that the boundary of the tumor was unclear and involved the sacrum, a complete resection would be difficult. Therefore, we administered neoadjuvant radiotherapy to the affected area at a dose of D_T_ 5000 cGy in 25 fractions to the planning target volume (PTV). After shrinkage of the tumor volume, the patient underwent complete extensive resection at 1 month after radiotherapy. Postoperative pathology confirmed that resection of a lesion measuring 7.5 cm × 4 cm × 3.5 cm achieved negative histological margins and indicated a classification of the specimen as a mesenchymal-derived malignant tumor involving the sacrum. Histologic examination of the resected tumor revealed undifferentiated pleomorphic spindle cells surrounding an area of geographic necrosis with frequent atypical mitosis. Microscopically, the morphology conformed to that of a high-grade spindle cell sarcoma consistent with UPS. The result from MDM2 amplification using fluorescence in situ hybridization was negative, and thus, lipogenesis on histology could be excluded (Additional file 1). The expression profile of the UPS tissue is described in Table 1, and representative images of staining tumor tissue are presented in Fig. 1.

**Table 1:** Expression profile of UPS tumor based on immunohistochemical staining of surgically resected tumor tissue

Positive	INI-1 (+), vimentin (+), S-100 (focally+), p53 (partially+), Bcl-2 (partially+), CD99 (+), calponin (sporadically+), Ki-67 (+, 15%), transducin-like enhancer of Split 1 (TLE1+), melan-A (focally weak+).
Negative	AE1/AE3 (−), desmin (−), CD31 (−), caldesmon (−), CK (−), EMA (−), ALK (−), SMA (−), CD117\c-kit (−), CD34 (−), MyoD1 (−), myogenin (−), CK/LMW (−), CK5/6 (−), 34βE12 (−), CAM5.2 (−), HMB45 (−), SOX10 (−), MITF (−).

**Fig. 2:**
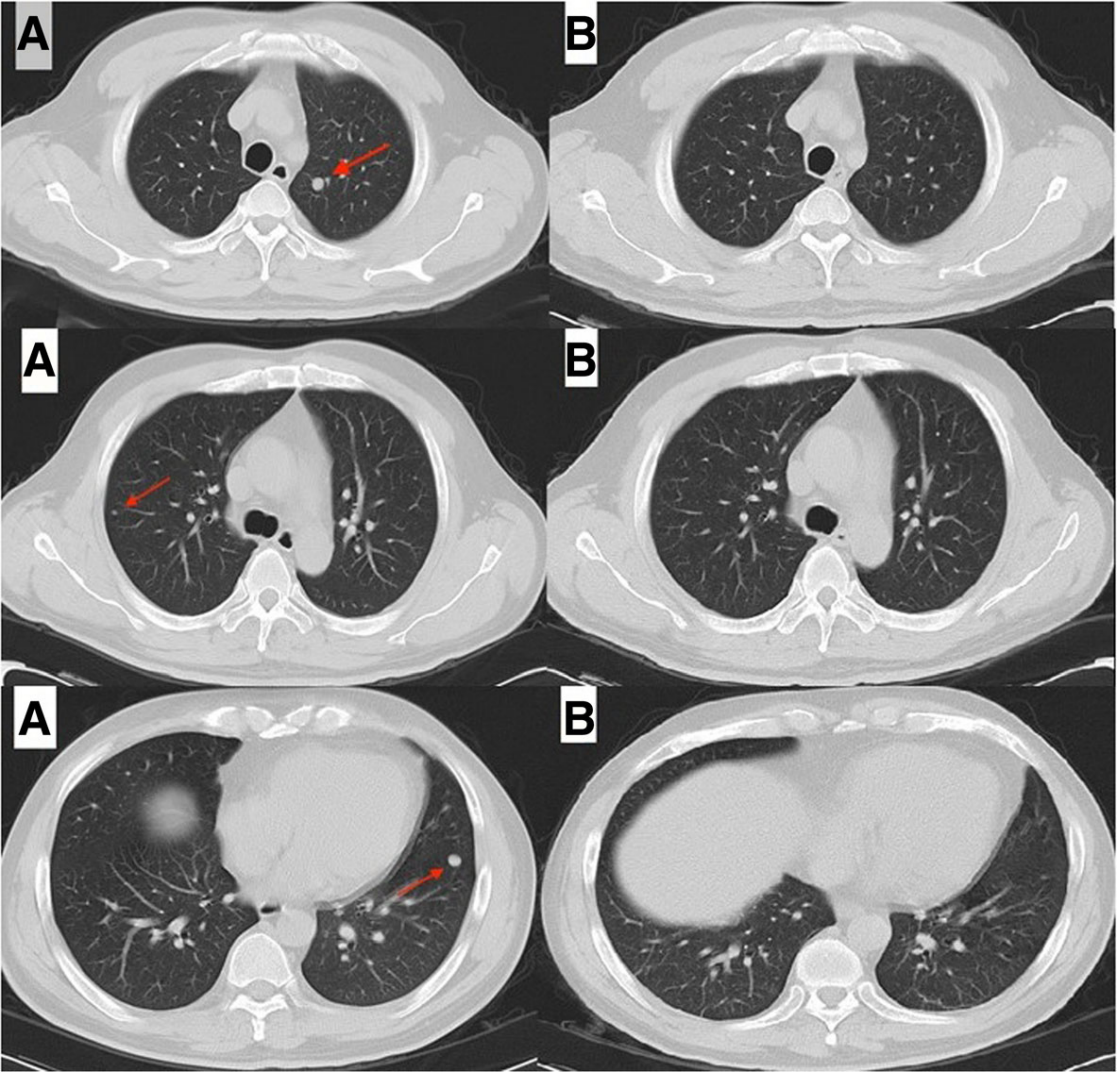
Chest CT images. (**a**) Follow-up chest CT images taken 3 months after surgery on January 10, 2017 demonstrated three new lesions (arrows) in the bilateral pulmonary region, before treatment with cizotinib. **b** Follow-up chest CT images taken on February 20, 2017 at 4 weeks after the initiation of oral crizotinib administration indicated improvement

**Fig. 3:**
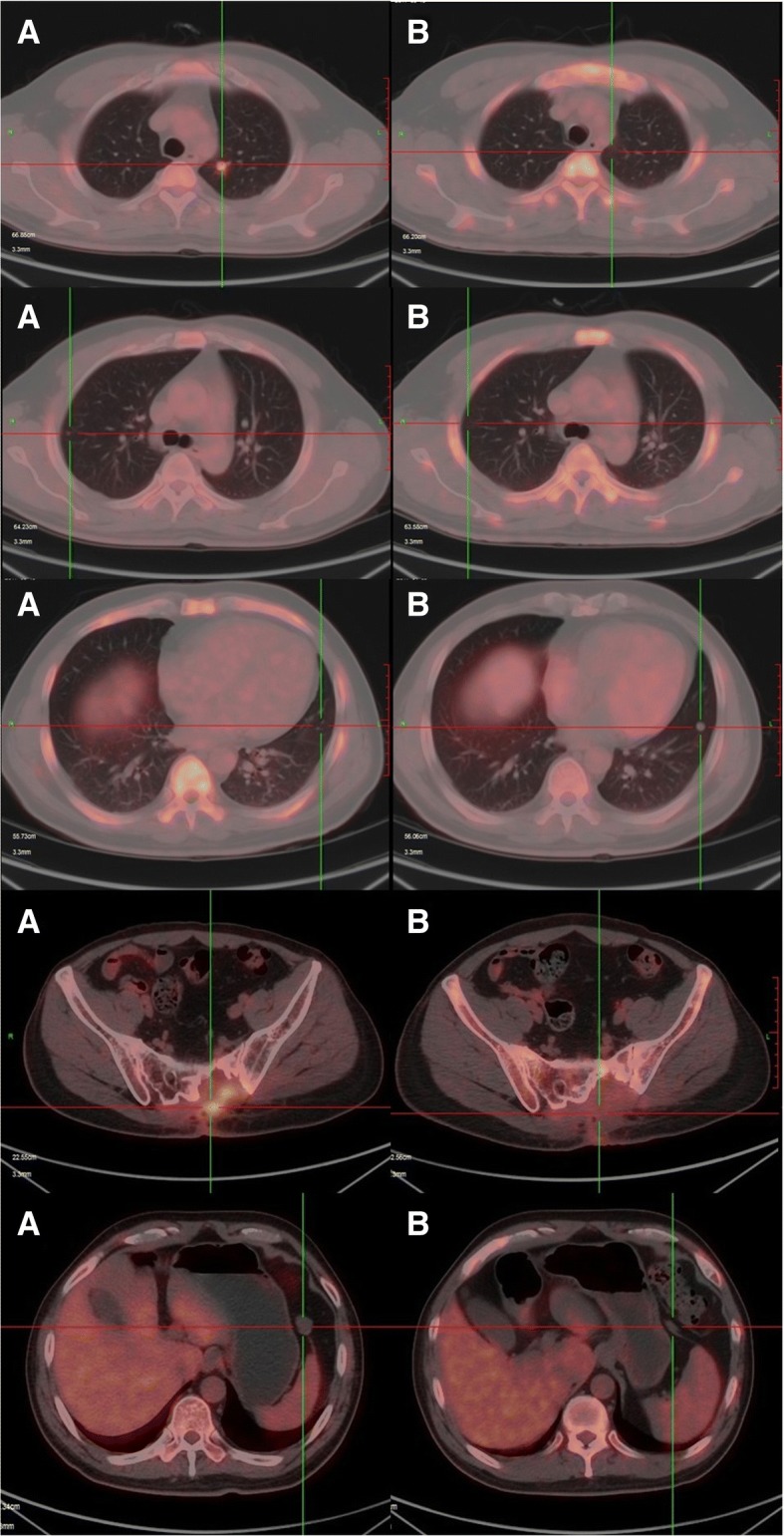
PET-CT images showing visible regression of the multiple metastases after 16 weeks of crizotinib monocherapy. **a** Follow-up PET-CT image taken on January 10, 2017 at 3 months after surgery showed hypermetabolic metastases in multiple regions, before the start of cizotinib treatment. **b** Follow-up PET-CT images taken on May 19, 2017 at 4 months after initiation of crizotinib showed near-CCR

A postsurgical MRI scan obtained 1 month after surgery showed postoperative changes and no obvious mass in the surgical area. The patient underwent adjuvant chemotherapy with liposomal doxorubicin and ifosfamide but had to discontinue chemotherapy after 2 cycles due to intolerance of grade 3 fatigue and grade 2 nausea. At 3 months after surgery, three new lesions were discovered in the bilateral pulmonary region on a routine follow-up CT scan (Fig. 2a). Further radiographic imaging with PET/CT showed hypermetabolic metastases involving the erector spinae of the left posterior sacral, fifth lumbar spine, sacrum, left ilium, and twelfth thoracic vertebra, accompanied by multiple lung lesions and a suspected metastasis adjacent to the spleen (Fig. 3a). At this stage, the patient refused further chemotherapy.

**Fig. 4:**
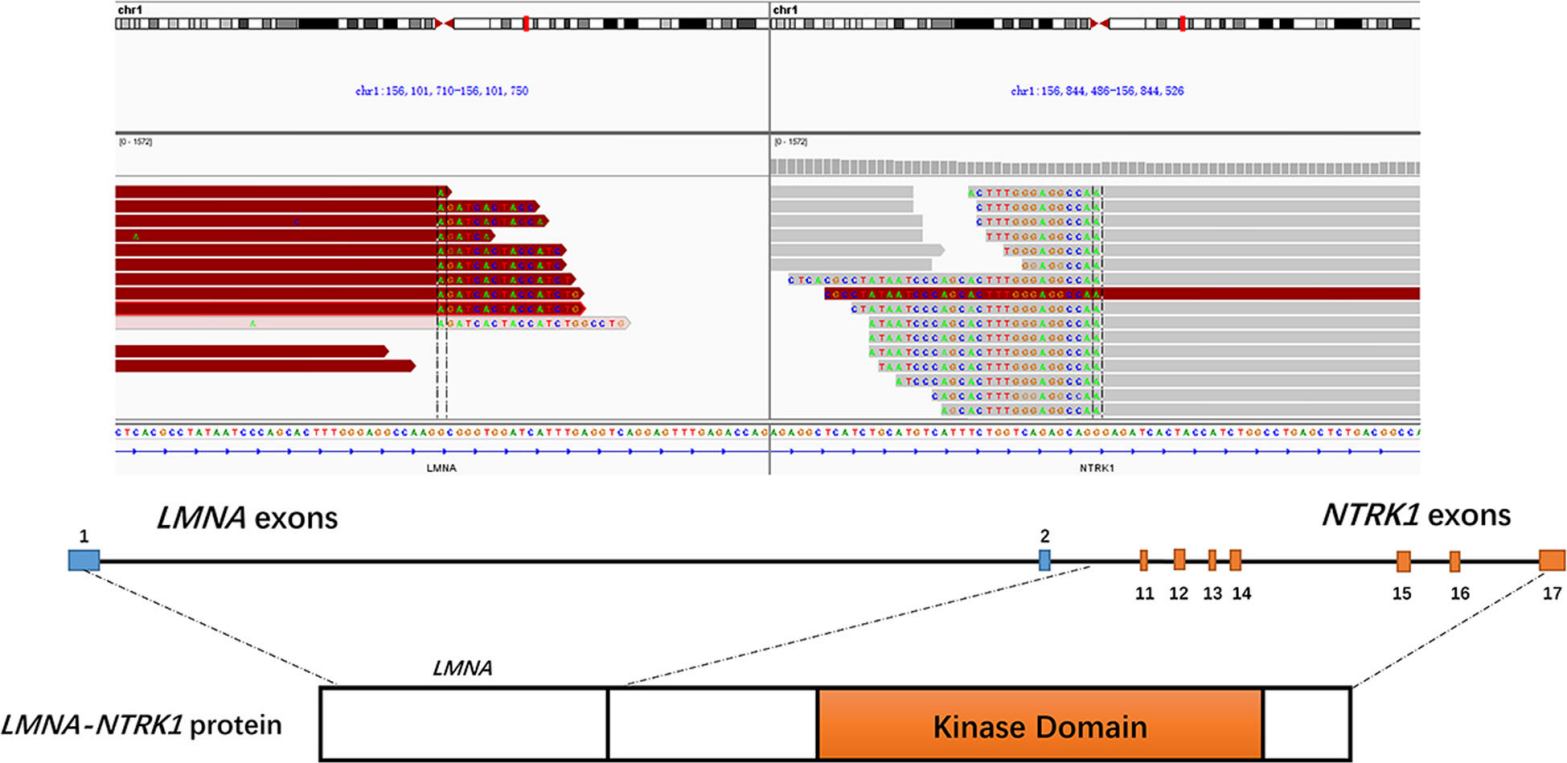
Schematic presentation of the LMNA–NTRK1 gene fusion. The fusion consisted of LMNA exons 1–2 followed by NTRK1 exons 11–17

With the standard therapeutic options exhausted, primary tumor tissue was subjected to DNA sequencing via next-generation sequencing (NGS) with an ILLUMINA Nextseq 500 (3DMedicines, Inc.). The MasterView 381 cancer-gene panel covered 4557 exons of 365 cancer-related genes and 47 introns of 25 genes frequently rearranged in 381 cancer-related genes (Additional file 2). The genomic DNA was extracted with the QIAamp DNA formalin-fixed paraffin-embedded tissue kit (Qiagen) following the manufacturer’s protocol and quantified with the Qubit™ dsDNA HS Assay kit (Invitrogen). Bioinformatics analyses involved analyzing the clipped reads, which can be extracted by the tag information of bam files, which mapped the individual reads to the reference human genome (hg19) with bwa aligner v0.7.12. Candidate reads that were discordant or aligned in the same direction were filtered. Read pairs with reads mapped to separate chromosomes or separated by a distance of over 2 kb on the same chromosome were kept for fusion detection at the probe level. Output rearrangements contained translocation, inversion, long deletion, etc. [5]. Through this profiling, a LMNA-NTRK1 gene fusion encoding exons 1–2 of lamin A/C and exons 11–17 of the NTRK1 gene was identified (Fig. 4), and the other unlisted genes were all wild-type. The sequencing results for the LMNA-NTRK1 gene fusion are presented in Additional files 3 and 4.

**Fig. 5:**
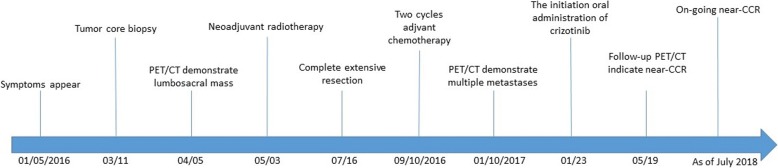
Timeline of the patient’s clinical course

After extensive discussion and consultation with the patient and his family, we initiated crizotinib therapy per os at 450 mg per day on January 23, 2017. One month later, chest CT scanning showed that all lesions in the bilateral lungs had almost disappeared, and the patient had achieved a near-complete clinical response (CCR, Fig. 2b). PET/CT imaging was repeated after 4 months of treatment and continued to show the same response to crizotinib therapy. PET/CT revealed that local FDG metabolism was slightly increased at the lesions of the fifth lumbar spine, sacrum, left ilium and left paraspinal muscle. However, with crizotinib treatment, the FDG metabolism was significantly reduced in comparison with that seen in the first PET-CT examination. The bilateral pulmonary nodules had disappeared, and the twelfth vertebra, which had shown osteolytic bone destruction, now showed signs of healing, with an increased density and a lower FDG metabolism. The volume of the left front nodule of the spleen was significantly reduced after treatment (Fig. 3b). A timeline of the treatment course is presented in Fig. 5. As of July 2018, clinical assessments in this patient showed an ongoing near-CCR of 18 months. In general, the side effects of oral administration of crizotinib at 450 mg per day were tolerable for the patient. During the course of treatment, the patient experienced grade 3 myelosuppression and grade 2 weakness, but myelosuppression could be alleviated with granulocyte colony-stimulating factor (G-CSF)-based supportive treatment.

## Discussion and conclusions

Approximately 5–15% of STS lesions cannot be differentiated by current molecular technologies or immunohistochemical criteria and are therefore classified as UPSs in an exclusion-based diagnosis [6]. The morphology of the primary tumor in the present case showed an ordered storiform pattern on hematoxylin and eosin (H&E) staining and progressively dedifferentiated to a highly pleomorphic tumor without definite true histiocytic differentiation. In addition, the tumor cells were mainly spindly with elongated, tapering nuclei. Considering also the findings on immunohistochemical staining after surgery, we finally confirmed a diagnosis of high-grade spindle cell UPS. The main pathology-based differential diagnosis among different potential histological entities was based on morphology as well as the expression profile of a panel of immunocytochemical markers. Before rendering the diagnosis of UPSs, the differential diagnoses that must be excluded include dedifferentiated liposarcoma, pleomorphic liposarcoma, pleomorphic leiomyosarcoma, pleomorphic rhabdomyosarcoma, high grade and epithelioid variant of myxofibrosarcoma, poorly differentiated carcinoma, and melanoma [7]. The diagnosis of primary UPS is made easier by extensive tumor sampling, evaluation of the overall morphologic pattern, careful searching for the best-differentiated area, and determination of the specific immunophenotype to evaluate a particular lineage of differentiation. In the present case, the initial diagnostic classification was difficult.

Current knowledge on UPSs suggests an aggressive clinical course, high incidence of recurrence and metastasis compared with other histologic STS subtypes [8]. Treatment with surgery only leads to poor rates of local control and even survival. To date, the clinical benefit of adjuvant chemotherapy and radiation remains unclear. More recently, genetic studies have contributed to an increased understanding of sarcomas and provided possible therapeutic advancements by identifying genetic markers of patients most likely to respond. In the present case, we identified a LMNA-NTRK1 fusion gene comprising exons 11–17 of the NKRT1 gene and exons 1–2 of LMNA gene in the patient’s tumor. The NTRK1 gene encodes tropomyosin receptor kinase A (TrkA), which is a membrane-bound receptor that, upon neurotrophin binding, undergoes autophosphorylation and activates members of the mitogen activated protein kinase (MAPK) pathway [9, 10]. The LMNA gene (localized at chromosome 1q22) encodes a key component of the nuclear lamina that is involved in nuclear assembly and chromatin organization. TrkA does not appear to be an oncogene, but gene fusions involving NTRK1 have been shown to be oncogenic, resulting in constitutive TrkA activation [11]. Activation of this receptor initiates several key downstream signal transduction cascades, including the MAPK, phosphatidylinositol 3-kinase (PI3K), and phospholipase C-γ (PLC-γ) pathways [12] as well as promotes phosphorylation of the AKT, ERK, and PLC-γ1 fusion proteins in vitro. Strong activation of the MAPK, PLC-γ1 and PI3K pathways can be inhibited by the NTRK1 inhibitor AZ-23 [13].

At present, no direct kinase inhibitors with NTRK1 fusions have been approved by the U.S. Food and Drug Administration. Doebele et al. [14] reported the case of a 41-year-old woman with an undifferentiated soft tissue sarcoma and lung metastasis harboring a LMNA-NTRK1 gene fusion who consented to treatment with the Trk inhibitor LOXO-101. Her tumors underwent rapid and substantial regression, with improvements in pulmonary dyspnea, oxygen saturation and reductions in plasma tumor markers. In another case of congenital infantile fibrosarcoma harboring a LMNA-NTRK1 gene fusion, a complete response to crizotinib therapy over 12 weeks was reported [15]. Crizotinib is a multi-active kinase inhibitor that blocks TrkA autophosphorylation and cell growth in cells expressing NTRK1 fusion proteins [11]. Notably, targeted crizotinib therapy is superior to standard chemotherapy in lung cancer patients with ALK fusions [16]. Based on the report of a minor response to crizotinib in a case of non-small cell lung cancer harboring a NTRK1 fusion as well as preclinical data [11], we started oral administration of crizotinib (450 mg QD) in the UPS patient described in this report. Over the follow-up period, the patient did not experience intolerable adverse effects from treatment and continued crizotinib monotherapy with no evidence of disease for more than 18 months as of July 2018. To our knowledge, this is the first case of UPS with a LMNA-NTRK1 gene fusion showing a durable response to crizotinib.

After screening a total of 1272 soft tissue sarcomas, Doebele et al. [14] identified five cases with a NTRK1 gene fusion, including three pediatric cases aged < 5 years and two adults. Thus, the detection rate for NTRK1 fusions in STS was less than 1% in their study. Haller et al [17] also reported four cases of sarcomas harboring NTRK1 gene fusions. The patients were two children aged 11 months and 2 years and two adults aged 51 and 80 years. The histomorphology in these cases was also described as characteristic spindle cell features, corresponing well to observations in the present case. These findings highlight the importance of further large research series with genetic testing of any sarcomatous neoplasm with similar histomorphology features for NTRK1 gene fusion and the application of such testing in the routine clinical diagnostic setting. The tumor regression and clinical response observed in the present case establishes that this LMNA-NTRK1 fusion may be a molecular driver of carcinogenesis in this patient and provides clinical validation of a molecular target in oncology. The oncogene driver may be the dominant factor in determining the response to targeted therapy, rather than the histologic subtype. We will continue following the clinical course of the patient to monitor the duration of the response, investigate how crizotinib has impacted the tumor, and track the potential development of treatment resistance.

In summary, this case provides robust evidence for the importance of molecular evaluation in cases of these rare but aggressive lesions and stresses the need for the development of drugs for better molecularly targeted STS treatment, especially when standard-of-care options have been exhausted or treatment options are unavailable.

## Additional files

Additional file 1: FISH result of MDM2 amplification. (PDF 299 kb)

Additional file 2: The MasterView 381 cancer-gene panel. (PDF 77 kb)

Additional file 3: LMNA BLAST. (PDF 42 kb)

Additional file 4: NTRK1 BLAST. (PDF 47 kb)

## Abbreviations

CCR: Complete clinical response
CK: Cytokeratin
CT: Computed tomography
G-CSF: Granulocyte colony-stimulating factor
H&E: Hematoxylin and eosin
MAPK: Mitogen-activated protein kinase
MFH: Malignant fibrous histiocytoma
MRI: Magnetic resonance imaging
NGS: Next-generation sequencing
PET/CT: Positron emission tomography−computed tomography
PI3K: Phosphatidylinositol 3-kinase
PLC-γ: Phospholipase C-γ
PTV: Planning target volume
SMA: Smooth muscle actin
STS: Soft tissue sarcoma
TrkA: Tropomyosin receptor kinase A
UPS: Undifferentiated pleomorphic sarcoma
