# Supplementary material for: A primary undifferentiated pleomorphic sarcoma of the lumbosacral region harboring a LMNA-NTRK1 gene fusion with durable clinical response to crizotinib: a case report
Source: BMC Cancer. 2018 Aug 22;18:842. doi: 10.1186/s12885-018-4749-z (PMC6106902; doi:10.1186/s12885-018-4749-z)
Supplement: Supplementary file 1 — FISH result of MDM2 amplification. (PDF 299 kb) [file 12885_2018_4749_MOESM1_ESM.pdf]

Formalin-fixed paraffin-embedded sections stained with MDM2/CEP12 FISH Probe.

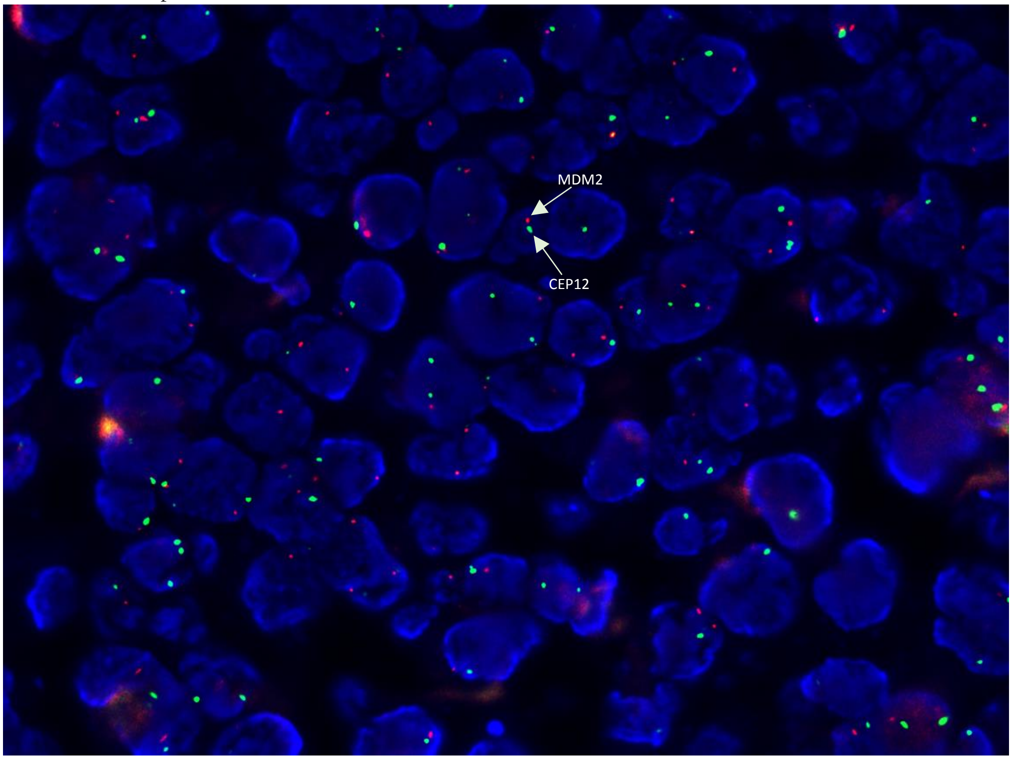

**signal number of MDM2 and CEP12 in 100 interphase cells**							
**Image#**	**Cell ID**	**MDM2**	**CEP-12**	**Image#**	**Cell ID**	**MDM2**	**CEP-12**
**16-17098C2**		3	3			3	3
		3	2			3	2
		3	3			2	2
		3	1			3	3
		4	2			4	3
		2	3			5	2
		1	1			2	1
		2	3			3	3
		2	2			2	2
		3	3			2	2
		4	3			2	2
		3	3			3	3
		2	1			3	3
		3	3			3	2
		3	2			5	3
		3	1			4	3
		2	2			2	2
		2	1			2	2
		2	1			2	1
		3	3			3	2

		5	3			3	3
		5	2			3	3
		2	1			5	3
		3	1			3	2
		4	2			2	2
		3	2			2	2
		2	3			4	3
		3	2			2	2
		3	1			2	1
		3	3			2	2
		3	2			3	3
		3	1			3	2
		2	1			3	3
		2	2			5	4
		2	1			4	3
		4	2			4	2
		1	2			2	2
		3	2			3	1
		1	1			3	3
		2	2			3	3
		4	2			2	2
		1	1			3	3
		1	1			3	2
		3	3			3	3
		3	3			4	3
		3	1			5	3
		3	2			2	2
		1	1			2	2
		4	4			3	2
		3	3			3	1
Total number of MDM2 signals		284		Average number of MDM2 signals		2,84	
Total number of CEP12 signals		218		Average number of CEP12 signals		2,18	
Totel Cell number		100					
**result**	**MDM2/CEP12=**	**1,30**					
	**MDM2/CEP12 ratio⩾2.0 means positive**						

Approximately 100 interphase cells were evaluated with each probe set. MDM2/CEP12 ratio⩾2.0 of tumor nuclei showing amplification was considered positive. In terms of our sample, the ratio was 1.3, that means MDM2 amplification status was negative, lipogenesis histology can be excluded.
