# Supplementary material for: A primary undifferentiated pleomorphic sarcoma of the lumbosacral region harboring a LMNA-NTRK1 gene fusion with durable clinical response to crizotinib: a case report
Source: BMC Cancer. 2018 Aug 22;18:842. doi: 10.1186/s12885-018-4749-z (PMC6106902; doi:10.1186/s12885-018-4749-z)
Supplement: Supplementary file 2 — The MasterView 381 cancer-gene panel. (PDF 77 kb) [file 12885_2018_4749_MOESM2_ESM.pdf]

The MasterView 381 cancer-gene panel

365 cancer-related genes

ABL1	ABL2	ACVR1B	ACVR2A	ADAM29	AKT1	AKT2	AKT3	ALK	AMER1	APC	AR	ARAF	ARFRP1
ARID1A	ARID1B	ARID2	ASXL1	ATM	ATR	ATRX	AURKA	AURKB	AXIN1	AXL	BAP1	BARD1	BCL2
BCL2L1	BCL2L2	BCL6	BCOR	BCORL1	BIRC5	BLK	BLM	BMX	BRAF	BRCA1	BRCA2	BRD4	BRIP1
BTG1	BTK	C11orf30	CARD11	CBFB	CBL	CCND1	CCND2	CCND3	CCNE1	CD274	CD79A	CD79B	CDC73
CDH1	CDK12	CDK4	CDK6	CDK8	CDKN1A	CDKN1B	CDKN2A	CDKN2B	CDKN2C	CEBPA	CHD2	CHD4	CHEK1
CHEK2	CIC	CRBN	CREBBP	CRKL	CRLF2	CSF1R	CSK	CSNK1A1	CTCF	CTNNA1	CTNNB1	CUL3	CXCR4
CYLD	DAXX	DDR1	DDR2	DICER1	DNMT3A	DOT1L	EGF	EGFR	EP300	EPHA2	EPHA3	EPHA5	EPHA7
EPHB1	ERBB2	ERBB3	ERBB4	ERCC1	ERG	ERRFI1	ESR1	EZH2	FAM135B	FAM46C	FANCA	FANCC	FANCD2
FANCE	FANCF	FANCG	FANCL	FAS	FAT1	FBXW7	FGF10	FGF14	FGF19	FGF23	FGF3	FGF4	FGF6
FGFR1	FGFR2	FGFR3	FGFR4	FGR	FH	FLCN	FLT1	FLT3	FLT4	FOXL2	FOXP1	FRS2	FUBP1
FYN	GABRA6	GATA1	GATA2	GATA3	GATA4	GATA6	GID4	GLI1	GLI2	GLI3	GNA11	GNA13	GNAQ
GNAS	GPR124	GRIN2A	GRM3	GSK3B	H3F3A	HCK	HGF	HNF1A	HRAS	HSD3B1	HSP90AA1	IDH1	IDH2
IGF1R	IGF2	IKBKE	IKZF1	IL7R	INHBA	INPP4B	IRF2	IRF4	IRS2	ITK	JAK1	JAK2	JAK3
JUN	KAT6A	KDM5A	KDM5C	KDM6A	KDR	KEAP1	KEL	KIT	KLHL6	KMT2A	KMT2C	KMT2D	KRAS
LCK	LIMK1	LMO1	LRP1	LRP1B	LYN	LZTR1	MAGI2	MAP2K1	MAP2K2	MAP2K4	MAP3K1	MAP4K5	MCL1
MDM2	MDM4	MED12	MEF2B	MEN1	MET	MITF	MLH1	MPL	MRE11A	MS4A1	MSH2	MSH6	MST1R
MTOR	MUTYH	MYC	MYCL	MYCN	MYD88	NEK11	NF1	NF2	NFE2L2	NFKBIA	NKX2-1	NOTCH1	NOTCH2
NOTCH3	NPM1	NRAS	NRG1	NRG3	NSD1	NTRK1	NTRK2	NTRK3	NUP93	PAK3	PALB2	PARK2	PAX5
PBRM1	PDCD1LG2	PDGFRA	PDGFRB	PDK1	PIK3C2B	PIK3CA	PIK3CB	PIK3CD	PIK3CG	PIK3R1	PIK3R2	PKD2	PLA2G1B
PLCG2	PMS2	POLD1	POLE	PPP2R1A	PRDM1	PREX2	PRKAR1A	PRKCI	PRKDC	PRSS8	PTCH1	PTEN	PTK2
PTK6	PTPN11	QKI	RAC1	RAD50	RAD51	RAF1	RANBP2	RARA	RB1	RBM10	RET	RICTOR	RNF43
ROCK1	ROCK2	ROS1	RPTOR	RUNX1	RUNX1T1	RXRA	SDHA	SDHB	SDHC	SDHD	SETD2	SF3B1	SIK1
SLIT2	SMAD2	SMAD3	SMAD4	SMARCA4	SMARCB1	SMO	SNCAIP	SOCS1	SOX10	SOX2	SOX9	SPEN	SPOP
SPTA1	SRC	SRMS	STAG2	STAT3	STAT4	STK11	STK24	SUFU	SYK	TAF1	TBX3	TCF7L2	TEK
TET2	TGFBR1	TGFBR2	TIE1	TNFAIP3	TNFRSF14	TNFSF11	TNK2	TOP1	TOP2A	TP53	TSC1	TSC2	TSHR
TYK2	U2AF1	VEGFA	VHL	WEE1	WEE2	WISP3	WT1	XIAP	XPO1	YES1	ZBTB2	ZNF217	ZNF703
ZNF750													

25 genes frequently rearranged in cancers

ALK	BCR	BRAF	BRCA1	BRCA2	BRD4	DDR2	ETV1	ETV4	ETV5	ETV6	FGFR1	FGFR2	FGFR3
MET	MYB	NOTCH2	NTRK1	NTRK2	PDGFRA	RAF1	RARA	RET	ROS1	TMPRSS2			
