# Supplementary figures and images for: A primary undifferentiated pleomorphic sarcoma of the lumbosacral region harboring a LMNA-NTRK1 gene fusion with durable clinical response to crizotinib: a case report
Source: BMC Cancer. 2018 Aug 22;18:842. doi: 10.1186/s12885-018-4749-z (PMC6106902; doi:10.1186/s12885-018-4749-z)

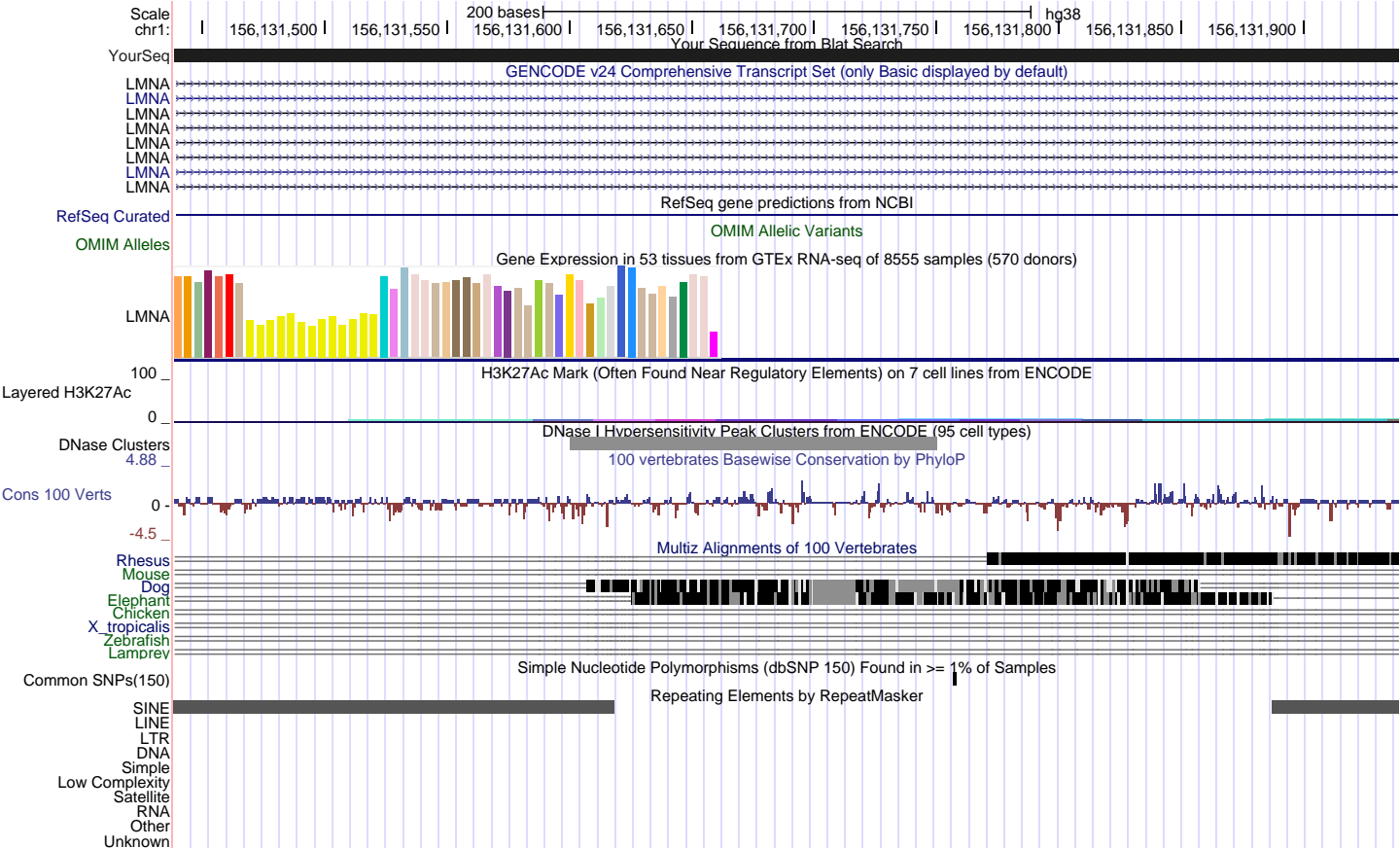

Supplement: Supplementary file 3 — LMNA BLAST. (PDF 42 kb) [file 12885_2018_4749_MOESM3_ESM.pdf]

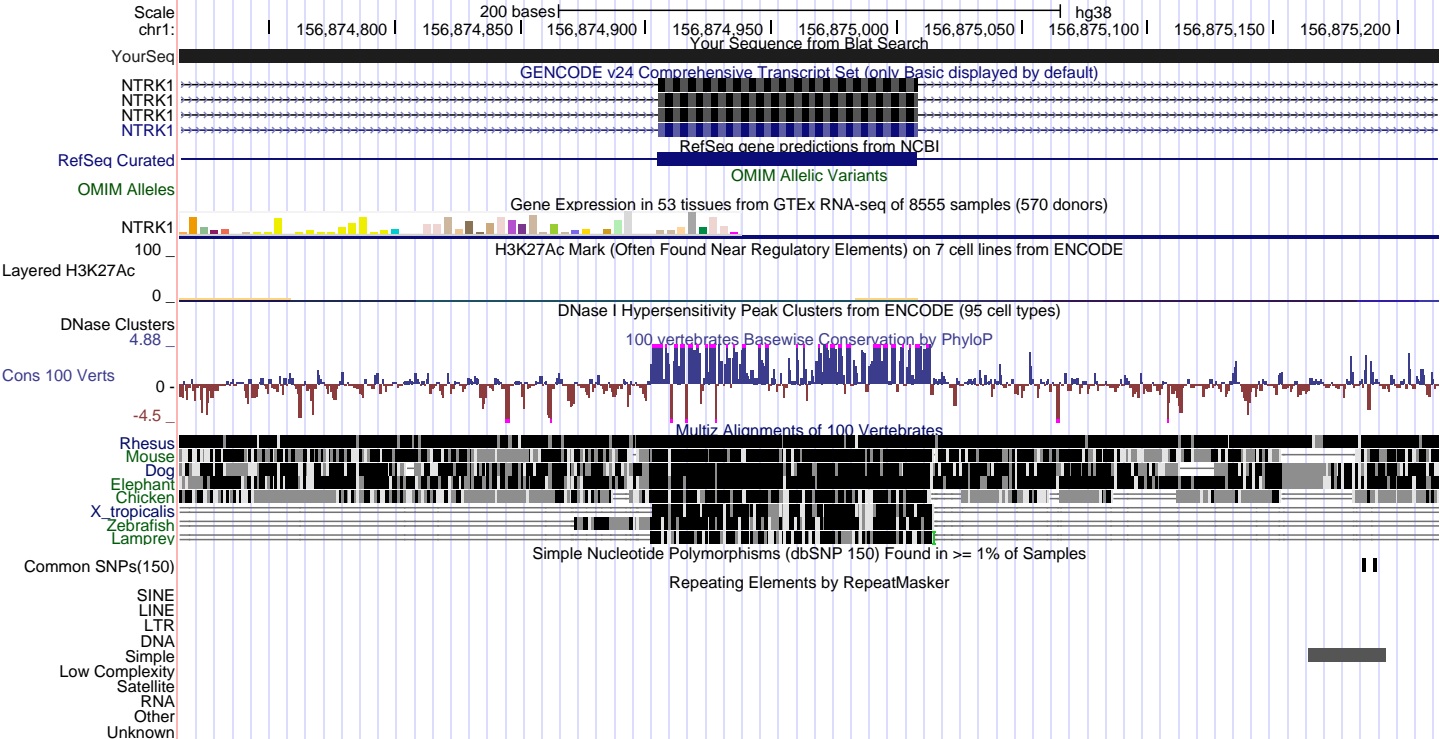

Supplement: Supplementary file 4 — NTRK1 BLAST. (PDF 47 kb) [file 12885_2018_4749_MOESM4_ESM.pdf]
